# Detection and Segmentation of Mature Green Tomatoes Based on Mask R-CNN with Automatic Image Acquisition Approach

**DOI:** 10.3390/s21237842

**Published:** 2021-11-25

**Authors:** Linlu Zu, Yanping Zhao, Jiuqin Liu, Fei Su, Yan Zhang, Pingzeng Liu

**Affiliations:** 1 College of Mechanical and Electronic Engineering, Shandong Agricultural University, Tai’an 271018, China; zulinlu@sdau.edu.cn (L.Z.); 2021110434@sdau.edu.cn (Y.Z.); 2020010028@sdau.edu.cn (J.L.); sufei@sdau.edu.cn (F.S.); 2 College of Information Science and Engineering, Shandong Agricultural University, Tai’an 271018, China; zhangyandxy@sdau.edu.cn

**Keywords:** Mask R-CNN, detection and segmentation, mature green tomato, mobile robot

## Abstract

Since the mature green tomatoes have color similar to branches and leaves, some are shaded by branches and leaves, and overlapped by other tomatoes, the accurate detection and location of these tomatoes is rather difficult. This paper proposes to use the Mask R-CNN algorithm for the detection and segmentation of mature green tomatoes. A mobile robot is designed to collect images round-the-clock and with different conditions in the whole greenhouse, thus, to make sure the captured dataset are not only objects with the interest of users. After the training process, RestNet50-FPN is selected as the backbone network. Then, the feature map is trained through the region proposal network to generate the region of interest (ROI), and the ROIAlign bilinear interpolation is used to calculate the target region, such that the corresponding region in the feature map is pooled to a fixed size based on the position coordinates of the preselection box. Finally, the detection and segmentation of mature green tomatoes is realized by the parallel actions of ROI target categories, bounding box regression and mask. When the Intersection over Union is equal to 0.5, the performance of the trained model is the best. The experimental results show that the F1-Score of bounding box and mask region all achieve 92.0%. The image acquisition processes are fully unobservable, without any user preselection, which are a highly heterogenic mix, the selected Mask R-CNN algorithm could also accurately detect mature green tomatoes. The performance of this proposed model in a real greenhouse harvesting environment is also evaluated, thus facilitating the direct application in a tomato harvesting robot.

## 1. Introduction

Tomatoes are one of the most extensively used fruit or vegetable crop in the world, with approximately 180 million tons of tomato fruits produced per year since 2016 [1,2]. Due to the desire of consumers for fresh tomatoes, especially the red color and firmness attributes [3], and to minimize transportation or other supply chain related injury, tomatoes are commercially picked at the mature green stage of ripening [4,5,6] because mature green tomatoes are still firm, have a longer shelf life, and can continue turning red even after being detached from the plant [7].

The picking of tomatoes is time-consuming and labor-intensive work. The automatic harvesting robots have become a research hotspot, the first step of automatic picking is fruit detection and localization on plants using computer vision [8,9,10]. Despite extensive research, fruit detection systems are still a weak link, which limits the commercial application of harvesting robots [11]. There are many research works demonstrating the effects of computer vision combined with machine learning for accurate tomato fruit detection [12,13,14,15,16]. For example, Yuanshen et al. propose the combination of AdaBoost classifier and color analysis to detect ripe tomatoes, the detection accuracy could reach 96% [13]. Peng et al. combine the feature color value and backpropagation neural network classification technique to detect the maturity levels (green, orange and red) of fresh tomatoes, the average accuracy is 99.31%; however, the tomato samples are taken to the lab after being picked, which cannot consider the disturbance of a complex environment [12]. The detection accuracy of the traditional machine learning method is mainly dependent on the handcrafted features (e.g., color, shape, texture, size), which is easily distorted by different lighting conditions, color similarity between fruits and plants, and occlusions [17,18].

With the development of deep learning, the convolutional neural networks (CNNs) are being increasingly used in the domain of fruit detection and yield estimation [19,20,21,22,23,24], such as Faster R-CNN based apple detection [24] and improved You Only Look Once (YOLO) based Mango detection [23]. For the detection of tomatoes, Sun et al. propose a modified Faster R-CNN model to detect and identify key organs of tomatoes, which could increase the detection mAP on flowers, green tomatoes and red tomatoes to 90.7% [25]. Liu et al. propose an improved tomato detection model based on YOLOv3 and use a circular bounding box for tomato localization, which contributes a 0.65% improvement on F1 score compared with the rectangular bounding box method [26]. Zhifeng et al. propose an improved YOLOv3-tiny method to detect mature red tomatoes, the F1-Score is 12% higher than the original YOLOv3-tiny model [27]. To avoid poor performance of the trained model caused by insufficient diversity of dataset, in the image acquisition process, the above studies consider different environment conditions including light, weather, angles, occlusions, and so on. However, the images are still captured manually, where the user operates like a monitor, directing the camera toward the object of interest. In this study, images are taken fully observable without any pre-selection, an automatic image acquisition method through a mobile robot traversing along the inspection route of the greenhouse is proposed. The prototype four-wheel-drive robot is equipped with a lifting platform and 4G wireless camera at the top, which could collect tomato images and then transmit to the cloud server through the LTE network.

The CNNs based fruit detection method can roughly calculate the position of the fruits using the bounding box, while cannot accurately extract contour and shape information. Therefore, the instance segmentation, which can provide geometric information for each fruit, is also necessary for accurate harvesting, especially for those overlapped fruits [28]. Yu et al. use Mask Region CNN (Mask R-CNN) for ripe strawberry detection, which demonstrates improved performance, especially for those overlapping and hidden fruits [29]. Kang et al. propose a Mobile-DasNet network and a segmented network for fruit identification, the accuracy is 90% and the instance segmentation accuracy is 82% [30]. Considering the color similarity among mature green tomatoes, leaves and branches, also the overlapping and occlusion problems under a complex environment, this paper also uses the Mask R-CNN network for the detection and segmentation of mature green tomatoes.

Considering that the tomato crops are located on large areas of the greenhouse, the harvesting robots must be mobile, the main aim of this study is to better facilitate the direct application of the tomato detection and segmentation method on harvesting robots. First, images are acquired through an RGB camera mounted on a mobile greenhouse robot, to be consistent with a real harvesting environment. Second, the detection and segmentation of mature green tomatoes in a greenhouse field experiment proved the effectiveness of trained Mask R-CNN to be used in harvesting robots.

## 2. Data Acquisition

### 2.1. Design of the Greenhouse Mobile Robot

**Figure 1:**
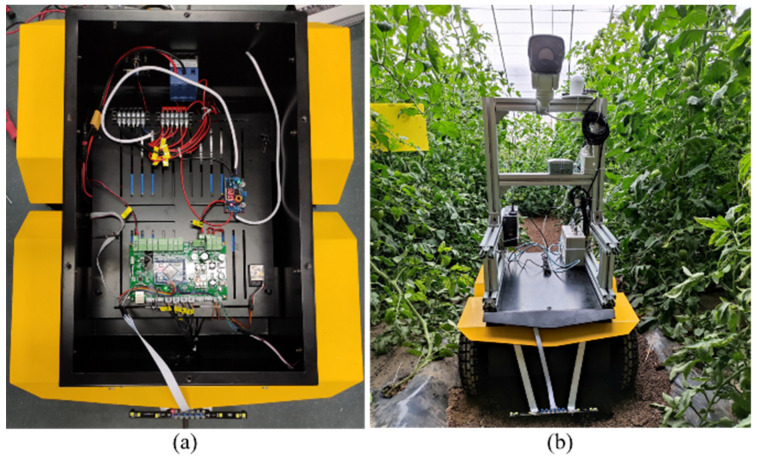
The (**a**) internal components and (**b**) working condition diagram of the greenhouse mobile robot.

This greenhouse mobile robot with a four-wheel-drive (4WD) platform has a dimension of 684 mm × 561 mm × 343 mm, the wheel diameter is 260 mm and the underpan height is 81 mm. Figure 1 shows the greenhouse working condition of the mobile robot. The robot has a 110 kg maximum pay load and maximum velocity of 1.8 m/s. The mobile robot is composed of the control unit, walking unit and navigation unit.

The STM32F407 microcontroller is used to control the whole system, which offers the performance of the Cortex™-M4 core running at 168 MHz. The 36V·20 AH lithium battery is used as the power supply, which can provide protection functions such as over charge, over discharge, short circuit and over current. The four direct-current motors (60AIM25C) are selected as the driving motors with a torque of 2 N·M, with a rated speed of 1000 r/min and a maximum speed of 1500 r/min. The 15-bit magnetoelectric encoder equipped with single-ring 32,768 pulse can accurately obtain the real-time speed information of the vehicle system and realize complete motion control.

**Figure 2:**
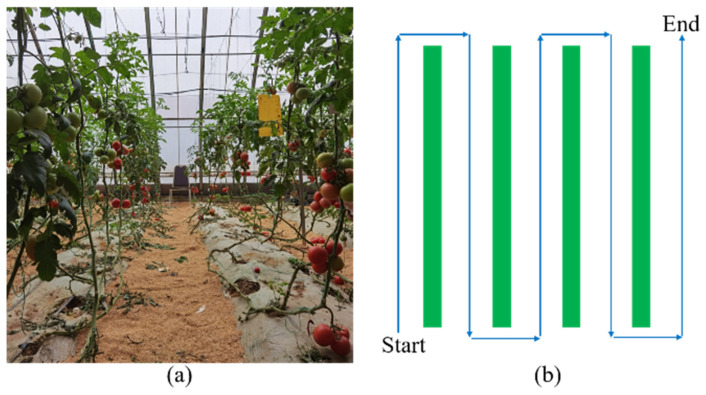
The (**a**)actual and (**b**) schematic diagram of the greenhouse path.

As shown in Figure 2, the navigation path of a greenhouse is relatively constant, thus the electromagnetic tracking method is used in this greenhouse mobile robot. First, the navigation signal wire is disposed under the ground between tomato rows, which is an enameled wire with a diameter of 1 mm, and an alternating current (frequency-20 kHz, amplitude-100 mA) flows through the wire. In addition, six electromagnetic sensors (each constitutes a resonance by a 10 mH inductor and 6.8 nF capacitance) are installed in front of the greenhouse mobile robot, with three on each side. Then, when the current in the wire changes according to a certain law, the magnetic field around the wire will also change, which induces a certain electromotive force in the coil. Finally, the signals obtained by the electromagnetic sensors are amplified and rectified by the LMV358 operational amplifier and transmitted to the analog-digital-converter interface of the STM32F407 microcontroller. According to the signal difference between two sides, the steering control is carried out to make sure the robot is moving along the navigation path.

### 2.2. Mature Green Tomato Image Acquisition

The 4G wireless RGB camera (Hykvision 3T46FDWD) is selected for image acquisition, which is mounted on the greenhouse robot. The mounting height of the camera is 90 cm from the ground, the vertical distance with the plant is around 50 cm. The horizontal and vertical field of this camera are 87.3 and 46.3°, respectively. The maximum image size obtained by this camera is 1920 × 1080 pixels. There are three main reasons to select this camera: first, the equipment head allows the user to remotely set the degree of camera rotation, to make sure a 50–160 cm plant field of view; then, it has a IP66 waterproofing grade, which is suitable for the humid working environment in greenhouse; third, it has a SIM card slot that supports 4G networks, the collected videos and images can be uploaded to the server through the high-speed 4G network.

**Figure 3:**
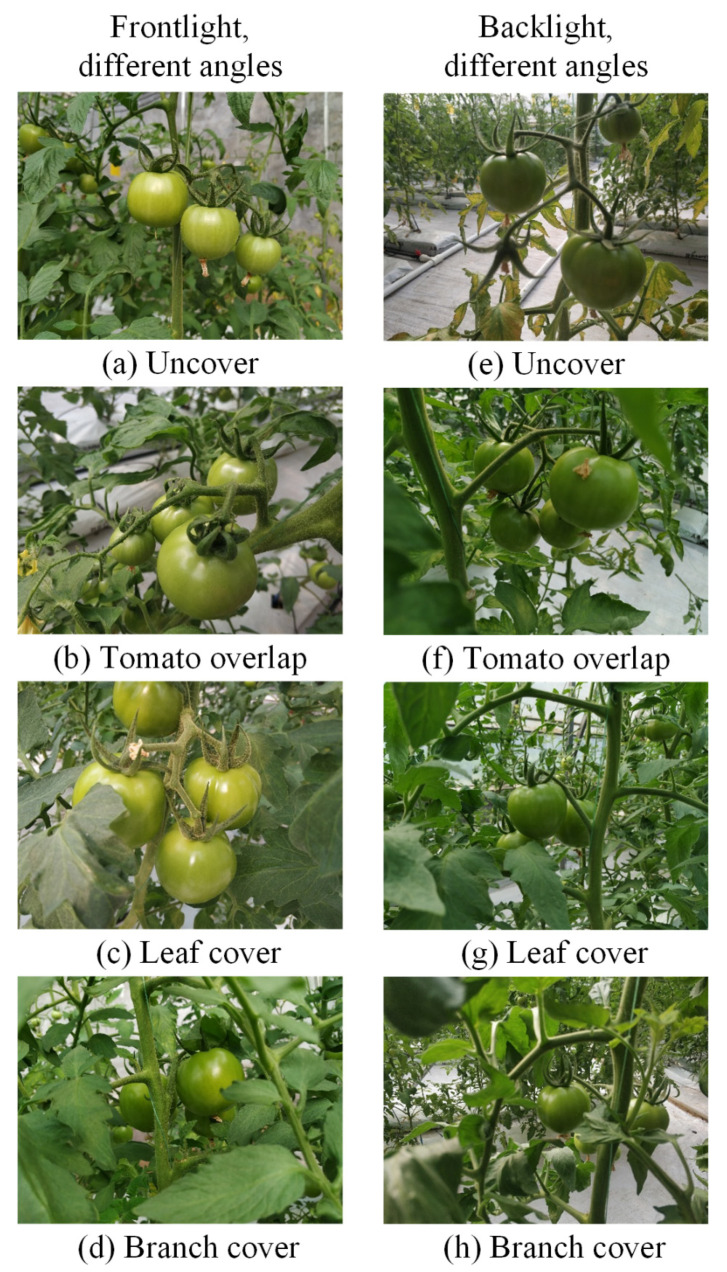
Images of mature green tomatoes from different angles under positive light and backlight conditions.

The greenhouse mobile robot can move along the navigation path and the camera shoots the video of tomatoes, which is uploaded to the server. Then, the video can be downloaded on the server side, thus the original pictures of tomatoes with 1920 × 1080 pixels are obtained by intercepting the video frames. In order to make sure the diversity of images used to train the Mask R-CNN network, tomato images are selected under both positive light and backlight conditions. As shown in Figure 3, each lighting condition includes images with no occlusion, fruit overlapping, leaf cover and branch cover.

## 3. Model Training and Loss Function

### 3.1. Image Labeling and Dataset Construction

**Figure 4:**
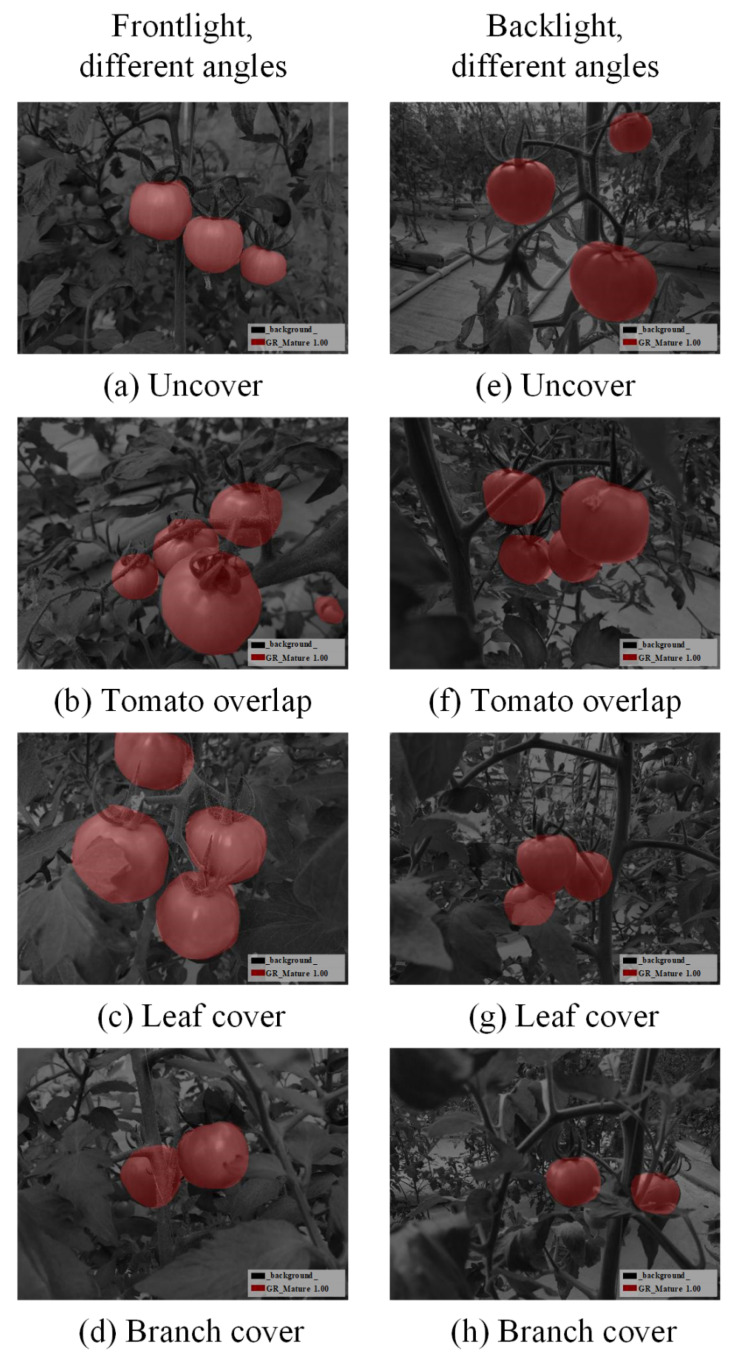
Visualization of manually annotated categories.

In order to improve the efficiency of image processing and prevent memory overflow during training, the original image is reduced to a uniform size (504 × 377 pixels). The whole image number used is 3180, where 2240 is randomly selected as the training set, 620 as the validation set, 320 as the test set, no data augmentation is used. Labelme (https://github.com/wkentaro/labelme) (accessed on 16 July 2021), an image annotation tool, is used to label the tomato images. In this paper, only one kind of tomato in green maturity stage was targeted, so the target category is one, and defined as GR_Mature_1.00, the rest are background categories defined as background. After labeling, the visualization effect of the category is shown in Figure 4.

### 3.2. Mature Green Tomato Detection and Segmentation Based on Mask R-CNN

**Figure 5:**
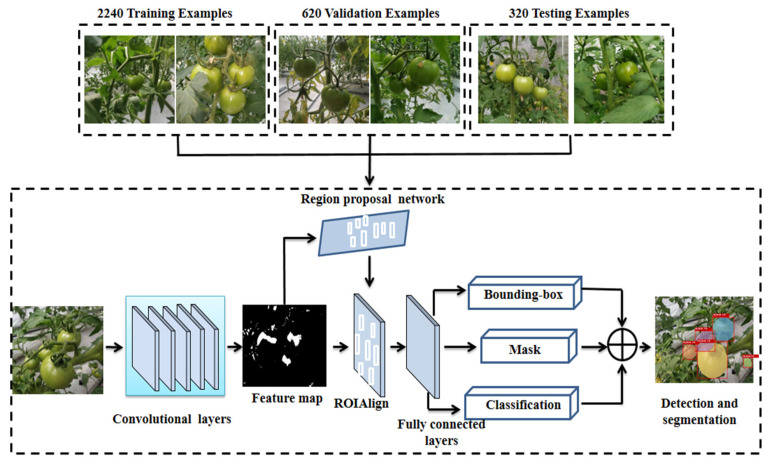
The structure of tomato detection and segmentation based on Mask R-CNN.

The flow of green mature tomato detection and segmentation based on Mask R-CNN is shown in Figure 5. First, the preprocessed image is input to the trained backbone network to extract the features of the tomato image. Then, the extracted eigenvalues are set to the region proposal network (RPN) to generate regions of interest (ROIs). Third, through the ROIAlign layer, the corresponding region is pooled into a fixed size in the feature map according to the position coordinates of the preselected box. Finally, the N-class classification, bounding box regression and mask generation are used to realize tomato detection and segmentation.

#### 3.2.1. Feature Extraction of Mature Green Tomato

The image feature extraction process simplifies the image into a feature map that can be recognized by the neural network through a convolution and pooling operation, which can filter out unnecessary interference information and reduce the calculation amount of the back layer network. The design of the feature extraction network is particularly important, which directly affects the accuracy and speed of image recognition. The performance of the model varies with the data sets; therefore, it is necessary to train and test the mature green tomato data set to select an appropriate backbone network. We compare the performance of the Mask R-CNN model with different backbone networks (ResNet50, ResNet50-FPN, ResNet101-FPN, ResNeXt101-vd-FPN and SENet154-vd-FPN). The evaluation indexes of model performance will be introduced in Section 4.2.

#### 3.2.2. Region Proposal Network

**Figure 6:**
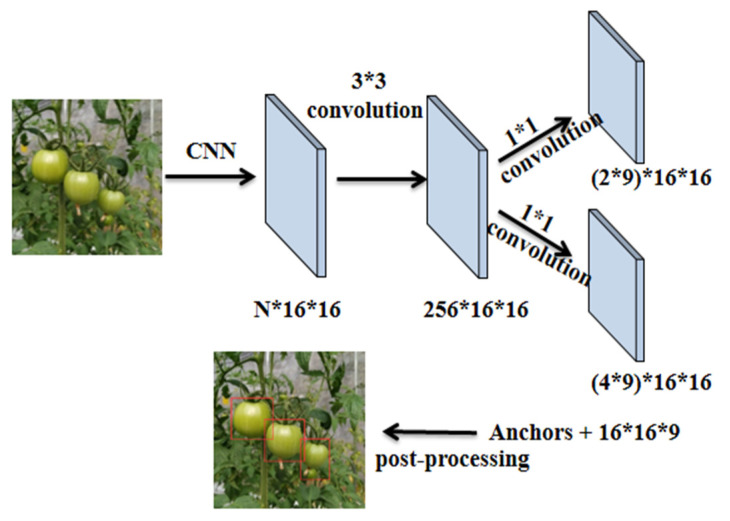
The process diagram of RPN.

The RPN is a full convolution network, whose input is the common feature graph extracted from the backbone network. Firstly, it passes through a 3 × 3 convolution layer, and then passes through two 1 × 1 convolution layers that are used for classification and regression respectively. In the classification layer, the softmax function is used to judge the foreground and background of the anchor points, and the anchor frames with Intersection over Union (IoU) greater than 0.7 are taken as the foreground and the anchor frames with IoU less than 0.3 used as the background. The offset of anchor frames is calculated by regression layer to obtain the target candidate frame of the mature green tomato. The whole process is shown in Figure 6.

#### 3.2.3. ROIAlign Layer

In Faster R-CNN, the role of ROI pooling is to pool the corresponding region into a fixed-size feature map according to the location coordinates of the preselected frame. Since the position of the preselected box is usually obtained by model regression, it is a floating-point number, while the size of the pooled feature map needs to be fixed. The ROI pooling operation has a twice quantization process, which will a cause region mismatch problem. To solve this, Mask R-CNN proposes a corresponding ROIAlign strategy. The quantization operation is cancelled, and the image values on the pixels whose coordinates are floating-point numbers are obtained by bilinear interpolation. The whole feature aggregation process is transformed into a continuous operation so that the corresponding features of each block can be found more accurately, and the features of each ROI are better aligned with the ROI region on the original image.

#### 3.2.4. Full Convolution Network Layer Segmentation

Mask R-CNN adds a branch for instance segmentation on the basis of Faster R-CNN, as shown in Figure 5, a convolution layer is added after ROIAlign to classify, bounding box regress and mask these ROIs. The instance segmentation is performed by the full convolution network (FCN), which can classify images at the pixel level, mainly including convolution and deconvolution. The image is convoluted and pooled to reduce the size of its feature map, then the deconvolution operation is used to increase the feature map. Finally, each pixel is classified to achieve accurate segmentation of the input image.

### 3.3. The Loss Function

The overall loss of Mask R-CNN includes two aspects: the loss of classification and regression operations by RPN and the training loss in the multi-branch predictive network. The total loss *L_final_* can be calculated by the following formula:

$$L_{final}=L_{RPN}+L_{Mul-Branch}$$

where, *L_RPN_* represents the training loss of the *RPN* and *L_Mul-Branch_* represents the training loss due to the branch structure.

In the training process of *RPN*, there are two things to perform for the generated anchors. The first one is to measure the probability of whether the anchor is the foreground or the background, and the second is to perform the preliminary coordinate correction for the anchors belonging to the foreground. Therefore, *L_RPN_* includes anchors classification loss (SoftMax Loss) and bounding box regression loss (SmoothL1 Loss). *L_RPN_* is calculated as follows:

(1) $$L_{RPN}=\frac{1}{N_{cls1}}\sum_{i}L_{cls}\left(p_{i},p_{i}^{*}\right)+\lambda_{1}\frac{1}{N_{reg1}}\sum_{i}p_{i}^{*}L_{reg}\left(t_{i},t_{i}^{*}\right)$$

*L_Mul-Branch_* is the sum of three-branch training losses (SoftMax Loss, SmoothL1 Loss and Mask Loss) in multi-branch predictive networks:

(2) $$\begin{array}{ll} L_{Mul-Branch} & =L\left(p_{i},p_{i}^{*},t_{i},t_{i}^{*},s_{i},s_{i}^{*}\right)\\ & =\frac{1}{N_{cls2}}\sum_{i}L_{cls}\left(p_{i},p_{i}^{*}\right)+\lambda_{2}\frac{1}{N_{reg2}}\sum_{i}p_{i}^{*}L_{reg}\left(t_{i},t_{i}^{*}\right)+\gamma_{2}\frac{1}{N_{mask}}\sum_{i}L_{mask}\left(s_{i},s_{i}^{*}\right) \end{array}$$

In the above formula, the constant N_*_ represents the number of corresponding anchor points or bounding boxes. The hyperparameters *λ*^∗^ and *γ*^∗^ balance the training losses of the regression and mask branches. Here, *s*^∗^ and *s* respectively represent the mask binary matrices from the prediction and ground-truth label. Classification loss *L_cls_*, regression loss *L_reg_* and mask loss *L_mask_* are derived from the following formulas:

(3) $$L_{cls}=-\log\left(p_{i}^{*}\times p_{i}\right)$$

(4) $$L_{reg}=R\left(t_{i}-t_{i}^{*}\right)$$

(5) $$L_{mask}=-\left(s_{}^{*}\log\left(s\right)+\left(1-s_{}^{*}\right)\log\left(1-s\right)\right)$$

where $p_{i}$ represents the classification probability of anchor *i* and $p_{i}^{*}$ represents the ground-truth label probability of anchor *i*; the variable $t_{i}$ represents the difference between the prediction bounding box and the ground-truth label box in four parameter vectors (the horizontal, the vertical coordinate value of the center point in the bounding box and the width and height of the bounding box); and $t_{i}^{*}$ indicates the difference between the ground-truth label box and the positive anchor, $s_{}^{*}$ and s respectively represent the mask binary matrices from the prediction and ground-truth label. *R* is a robust loss function (smooth *L*_1)_.

(6) $${\mathrm{smooth}}_{L_{1}}\left(x\right)=\left\{ \begin{array}{ll} 0.5x^{2} & \mathrm{if}\;\left|x\right|<1\\ \left|x\right|-0.5 & \mathrm{otherwise} \end{array}\right.$$

For each ROI, the mask branch has k*m*m dimension output, that is, k masks with m*m size are coded, and each mask has k categories. Since only tomato in green maturity stage was targeted, the value of k is 1. For each pixel, the sigmoid function is used to calculate the relative entropy, and the average binary cross-entropy loss function Lmask is obtained, which can effectively avoid inter-class competition.

### 3.4. Experiment Setup

In this study, the experiments are conducted on the AI studio platform (https://aistudio.baidu.com) (accessed on 20 August 2021). This platform provides NVIDIA TeslaV100 and 16 GB video memory for computing power configuration. The maximum training iteration number of the model is set to be 20,000, and the batch size is set to be 1 via trial-and-error. The trained model is evaluated once by the validation set every 200 iterations.

**Figure 7:**
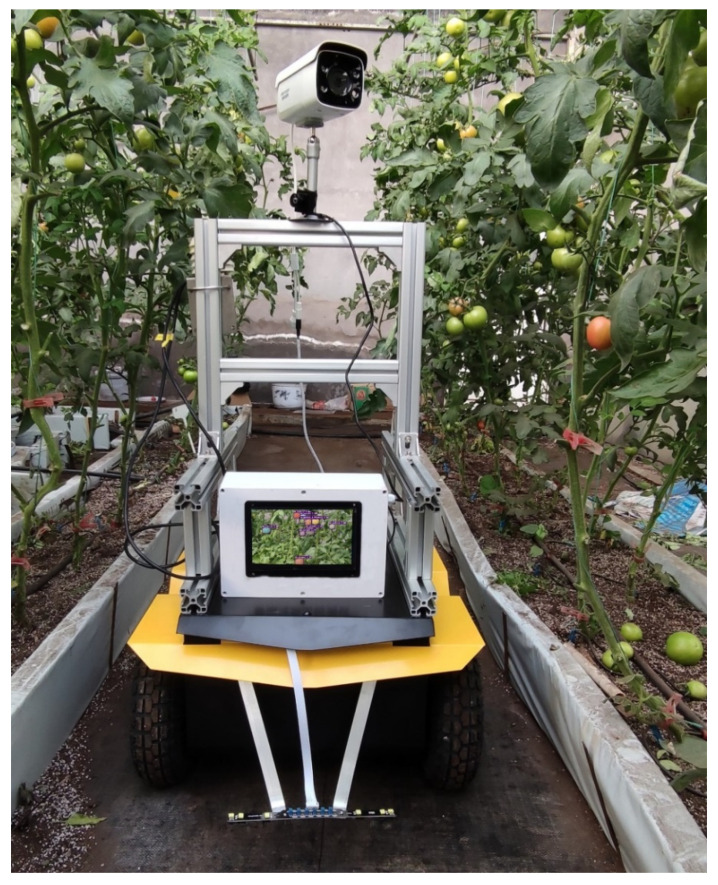
Model performance evaluation experiment in greenhouse field environment.

A series of experiments are conducted to evaluate the performance of the proposed method. The 320 test set is used to verify the reasoning and prediction performance of the trained model under different light, occlusion and angles conditions. To evaluate the performance of the proposed model in a real harvesting environment, the detection and segmentation experiment is also done in the tomato greenhouse of Shandong Agricultural University. As shown in Figure 7, the laptop computer with trained model is placed inside the greenhouse mobile robot, images collected by the camera are input into the model, and then the detection and segmentation results of mature green tomatoes can be displayed on the screen. By adjusting the angle of the camera, the number of tomatoes contained in one image was different, thus we could perform a data imbalance test.

## 4. Results

### 4.1. Model Performance Evaluation Indexes

Based on the COCO dataset evaluation, the F1-Score (Equation (7)) calculated from the precision and recall index is used to evaluate the accuracy of the model, and F1-Scorebbox and F1-ScoreMask represents the F1-Score of bounding box and mask, respectively.

(7) $$F1-Score=2\cdot \frac{precision\cdot recall}{precision+recall}$$

The FPS (frame per second) is the number of images processed per second is used to quantify the computational speed of the model. Considering the trade-off between accuracy and speed, the combined index is defined to quantify the performance of Mask R-CNN model.

(8) $$Index=\omega_{1}\cdot perunit\;FPS+\omega_{2}\cdot F1-Score_{bbox}+\omega_{3}\cdot F1-Score_{Mask}$$

Since F1-Score is a value between 0 and 1, the FPS index is also transformed into per-unit value, i.e., perunitFPS. The weights for different index items are set as $\omega_{1}=0.2$, $\omega_{2}=0.4$, and $\omega_{3}=0.4$ according to the accuracy and speed requirement in this study.

### 4.2. Selection of Backbone Network

The detection and segmentation of mature green tomatoes is trained based on different backbone networks, i.e., ResNet50, ResNet50-FPN, ResNet101-FPN, ResNeXt101-vd-FPN and SENet154-vd-FPN. The values of performance evaluation indexes with different backbone networks are shown in Table 1. Compared with other backbone networks, the reasoning time of ResNet50, ResNeXt101-FPN and SENet154-vd-FPN is longer (smaller FPS value), so reasoning and prediction will consume more time in the actual deployment, which cannot meet the real-time requirements of image detection and segmentation. ResNet50-FPN performs best for this customized mature green tomato dataset, therefore, which is selected as the backbone network of Mask R-CNN in this study.

### 4.3. Model Performance on the Test Set

**Figure 8:**
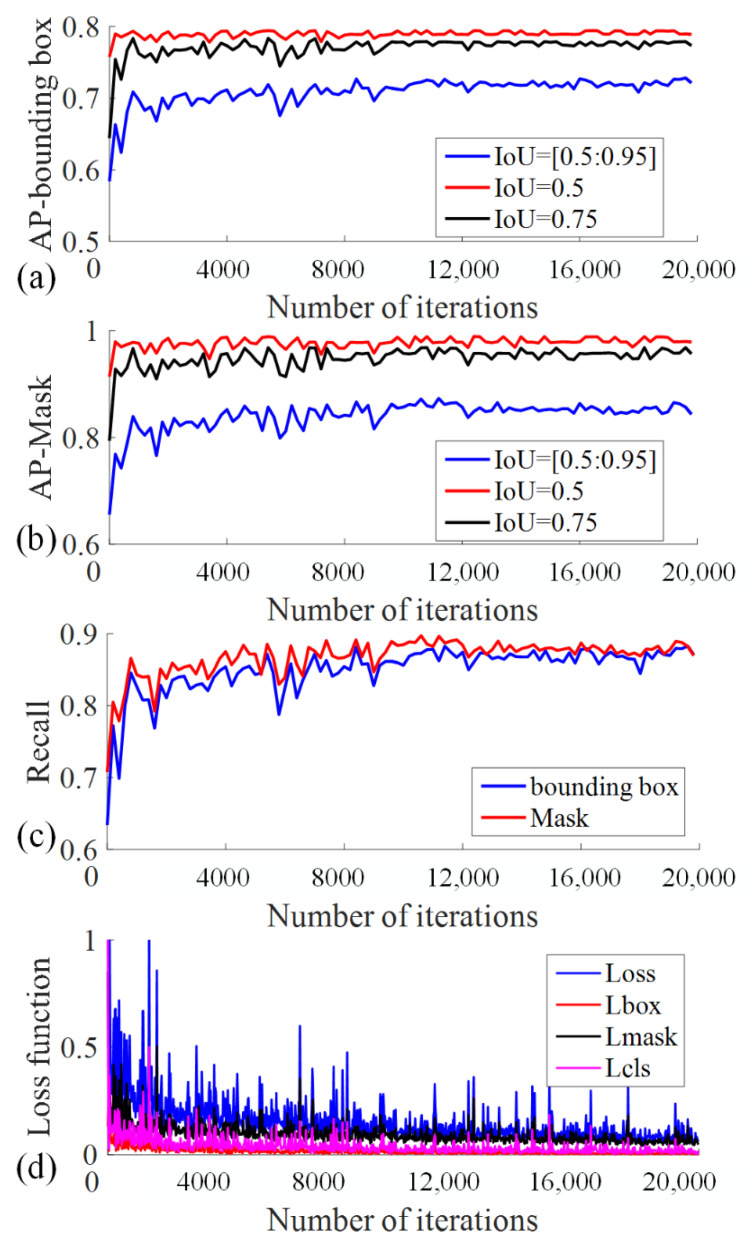
The performance evaluation index of Mask R-CNN model with ResNet50-FPN as the backbone network.

The model performance on the test set with ResNet50-FPN as the backbone network over different IoUs are shown in Figure 8. When the IoU increases from 0.5 to 0.95 (IoU = [0.5:0.95]), the AP value of the model increases gradually and then tends to be stable with the increase of training iterations; the loss function value first decreases and then tends to be stable, and the model converges normally. When the IoU = 0.5, the trained model has the highest accuracy, the F1-Score of bounding box and mask are both larger than 92%.

**Figure 9:**
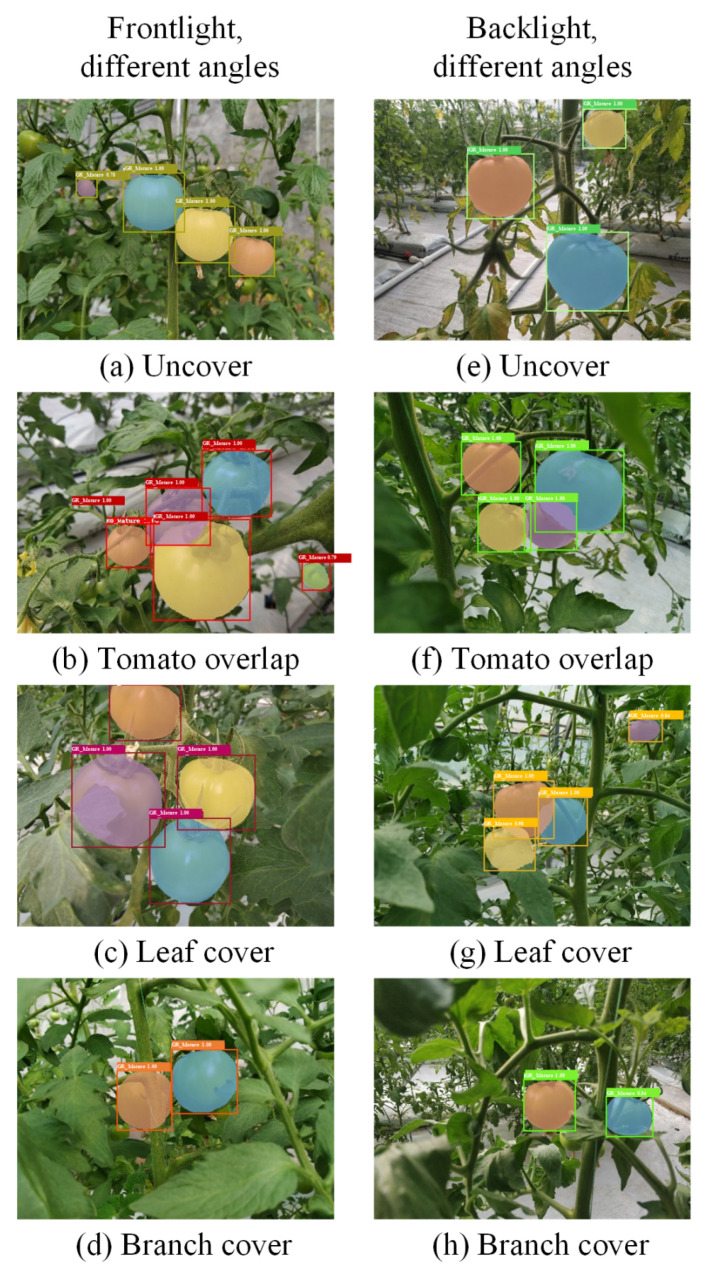
The detection and segmentation results of mature green tomatoes in different states.

Figure 9 presents the detection and segmentation performance on one test set, the same image as Figure 3 is given as an example. This model can detect and segment the mature green tomato fruit area, especially for those images with fruit overlapping, leaf occlusion, branch occlusion and other complex states of the tomato where traditional image detection algorithms are difficult to solve.

### 4.4. Model Performance in the Greenhouse Field Environment

**Figure 10:**
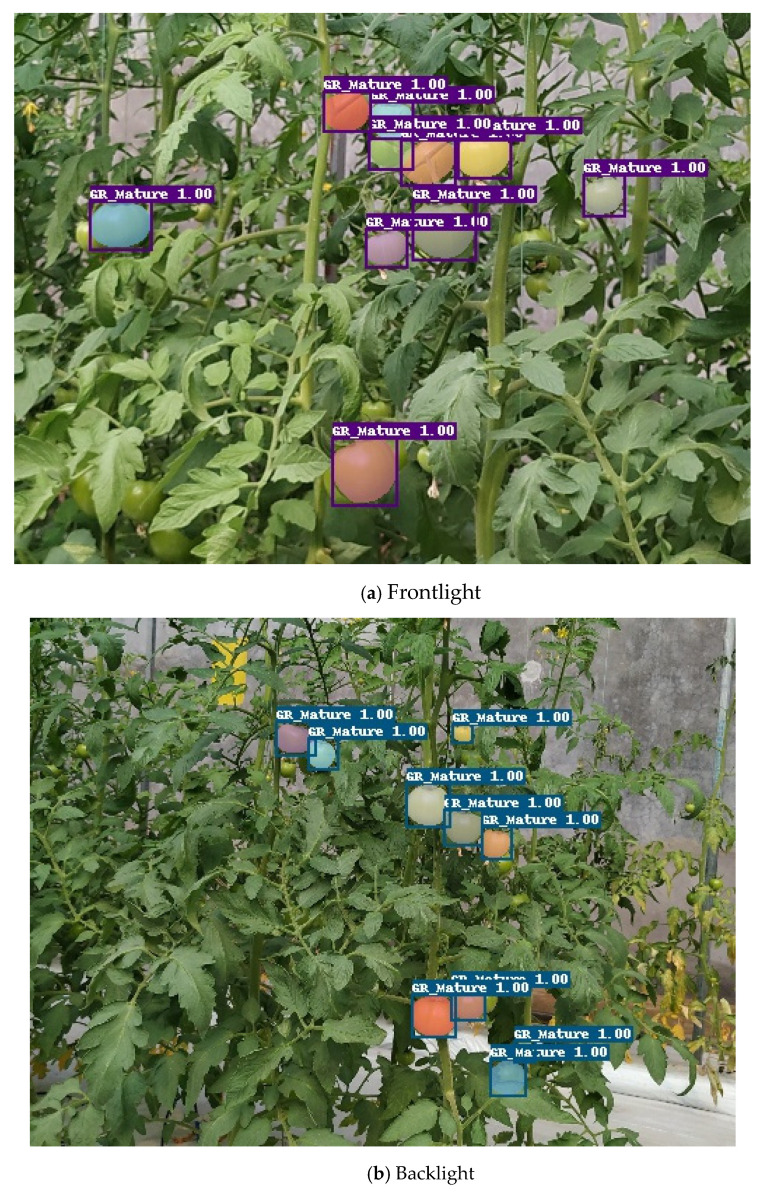
Results of the data imbalance test on mature green tomatoes.

By adjusting the camera angle, the distance between the camera and collected plants could vary from 50 cm to 160 cm, thus the number of tomatoes in one image varies from 3 to 14. Then, randomly select 15 images to perform manual detection. The results comparison with Mask R-CNN are shown in Table 2. Tomatoes in these images are classified to unshaded, lightly shaded and shaded more than 50% conditions. The number of tomatoes unshaded and lightly shaded is 101, among them 98 tomatoes are recognized by the trained model. The number of tomatoes shaded more than 50% is 21, 15 tomatoes are recognized. Thus, the total detection accuracy of Mask R-CNN is 92.6%. For tomatoes shaded more than 50%, the detection accuracy is only 71.4%, mainly because the pixels are less and the features are not obvious, which makes it difficult for the model to extract features of tomatoes. Besides, tomatoes under high shading conditions are always small objects, which also makes detection hard. In Figure 10, the detection and segmentation performance under frontlight and backlight conditions are given as an illustration.

## 5. Conclusions and Discussion

On-plant detection and segmentation of tomatoes is an important process of harvesting robots; current studies mainly include two kinds of methods, i.e., pixel-wise semantic segmentation deep learning algorithms and classical color-based segmentation. Huang et al. use user-collected RGB images and Mask R-CNN with ResNet-101-FPN backbone network to realize detection and segmentation of cherry tomatoes, and the average accuracy of 20 test images is 98.00% [31]. Afonso et al. use four RealSense cameras mounted on a trolley moving along the heating pipes to realize automatic image collection, the sensors need to be close to the fruit, with a distance roughly at 0.5 m [32]. However, in order to minimize the effects of lighting conditions, all images are collected at night. The Mask-RCNN with RestNext-101 backbone network is used to realize detection and segmentation of both red and green tomatoes, with F1-Score being 0.93 and 0.94 for these two types of fruits.

Tenorio et al. use an RGB camera coupled to a pipe rail trolley to automatically collect images of cherry tomato clusters, where the distance between the camera and the fruits is around 1 m [33]. Then, the MobileNetV1 CNN is used to detect cherry tomato clusters, with an accuracy of 95.98%, after that, the improved color space segmentation works better for red tomato clusters, while not so accurate for some mixed and green clusters. Benavides et al. use an RGB camera located perpendicular to the soil face and with a distance of 20–30 cm from the plant to collect images of beef and cluster tomatoes [34]. The Sobel operator is used to detect the tomato edge, then color-based and size-based segmentation, and finally realize the detection of ripe tomatoes and their peduncles in the foreground (not occluded) and segmentation from the rest of the image elements.

In this study, in order to maximum simulate the working condition of harvesting robots, images are automatically collected by an RGB camera mounted on a mobile robot, which could traverse along the greenhouse day and night. The Mask R-CNN with RestNet-50-FPN backbone network is used to detect and segment mature green tomatoes, with F1-Score being 0.9284. Table 3 shows the detailed comparison with existing studies.

In deep learning, the identification performance increases logarithmically with the increase of training and validation set number [35,36]; therefore, we also use other partitioning between training (training set and validation set) and testing number. Results show that only when the ratio of training is larger than 70% (i.e., the training set number is larger than 1272), the F1-Score of bounding box could reach 0.834, which is relatively satisfactory. However, when the ratio of training is 50% (i.e., the training set number is 318), the F1-Score of bounding box is only 0.421. However, well annotated datasets can be time-consuming, laborious and not reproducible, especially for classification tasks with large numbers of classes or with fine-grained classes. Even for the labelling of mature green tomatoes in this manuscript, which does not require expert knowledge, a skilled person can only label about 200 images per day. One approach to overcome this drawback is to apply a semi-supervised-based deep neural network, which is used to transfer knowledge from the labeled training data to unlabeled ones, which proved to be beneficial [37,38,39]. Images acquired in this study are only obtained from one greenhouse. Additional images from different kinds of greenhouse will be acquired to train a more robust model, which will require a more laborious labelling work; the semi-supervised methods could be referenced.

Tomatoes are commercially picked at the mature green stage due to the market requirement on fruit freshness, firmness and transportation-related injury [4,5,6]. Considering the color similarity between mature green tomatoes and environment, overlap among fruits, cover by leafs and branches, this paper proposes to use Mask R-CNN for the detection and segmentation of mature green tomatoes. Compared to laboratory experiment conditions, the greenhouse environment can be more challenging for image analysis, which can limit the placement of location of a camera and its field of view. Can we accurately detect and segment mature green tomato fruits in real harvesting conditions? The specific work is summarized as follows:

In order to make sure the diversity of a mature green tomato dataset, and simulate the automatic harvesting condition, this study proposes an automatic image acquisition method. The greenhouse mobile robot is designed to traverse along the navigation path by the electromagnetic tracking method and shoot the video of tomatoes in a greenhouse, the image dataset is obtained by intercepting the video frames.

The detection and segmentation of mature green tomatoes is realized based on the Mask R-CNN model. A total of 2240 tomato images are selected as a training set to train the Mask R-CNN network model, 620 images as a verification set and 320 images as a test set. Simulation results prove the effectiveness of this method, especially for those images with fruit overlapping, leaf occlusion, branch occlusion and other complex states. When IoU is 0.5, the model performance is the best, the F1-Score of bounding box and mask region for test set both reach 92.0%.

This method can significantly improve the detection and segmentation performance of mature green tomatoes in the complex greenhouse environment, which will lay a foundation for the accurate identification of mature green tomatoes for harvesting robots and provide technical support for the development of green fruit and vegetable automatic picking.

## Figures and Tables

**Table 1:** Evaluation index values for mature green tomato dataset under different backbone networks.

Backbone Network	FPS	F1-Score_bbox_	F1-Score_Mask_	Index
ResNet50	5.77	0.9336	0.9241	0.8531
ResNet50-FPN	26.10	0.9290	0.9284	0.9430
ResNet101-FPN	19.53	0.9265	0.9300	0.9135
ResNeXt101-vd-FPN	9.34	0.9302	0.9323	0.8710
SENet154-vd-FPN	3.49	0.9318	0.9345	0.8465

**Table 2:** Recognition performance comparison between the trained Mask R-CNN model and manual detection.

Samples	Number of Mature Green Tomatoes by Manual			Number of Mature Green Tomatoes Identified by Mask R-CNN			Recognition Accuracy/%
	Unshaded/Lightly Shaded	Shaded More Than 50%	Total	Unshaded/Lightly Shaded	Shaded More Than 50%	Total	
1	6	1	7	6	1	7	100
2	3	0	3	3	0	3	100
3	7	2	9	7	1	8	88.9
4	7	1	8	7	1	8	100
5	9	3	12	9	2	11	91.7
6	4	0	4	4	0	4	100
7	5	1	6	5	0	5	83.3
8	3	0	3	3	0	3	100
9	8	2	10	8	1	9	90
10	6	2	8	6	2	8	100
11	10	3	13	9	3	12	92.3
12	9	2	11	9	1	10	90.9
13	7	1	8	6	1	7	87.5
14	6	0	6	6	0	6	100
15	11	3	14	10	2	12	92.9
Total	101	21	122	98	15	113	92.6

**Table 3:** Scientific studies in tomato detection and segmentation based on image analysis.

Author	Method	Sensor	NO. Images	Reported Metrics
Huang et al., 2020 [31]	Mask R-CNN with ResNet-101-FPN	RGB camera	900 images with data augmentation	Detection accuracy of cherry tomato is 98%
Afonso et al., 2020 [32]	Mask R-CNN with ResNext-101	4 RealSense cameras mounted on a pipe rail trolley	123 images without data augmentation	F1-Score of red tomato is 0.93, and green tomato is 0.94
Tenorio et al., 2021 [33]	MobileNetV1 CNN for detection & color space segmentation	RGB camera mounted on a pipe rail trolley	254 images with data augmentation	Detection accuracy of cherry tomato cluster is 95.98%
Benavides et al., 2020 [34]	Sobel operator for detection, color-based segmentation and size-based segmentation	RGB camera located perpendicular to the soil surface	175 images	Detection accuracy of beef tomato 90%, and cluster tomato is 79.7%
Proposed	Mask R-CNN with ResNet-50-FPN	RGB camera mounted on a mobile greenhouse robot	3180 images without data augmentation	F1-Score of mask for green tomato is 0.9284

## Data Availability

Not applicable.
